# Does Changeover by an Experienced Open Prostatic Surgeon from Open Retropubic to Robot-Assisted Laparoscopic Prostatectomy Mean a Step Forward or Backward?

**DOI:** 10.1155/2013/768647

**Published:** 2013-01-21

**Authors:** Michael Musch, Ulla Roggenbuck, Virgilijus Klevecka, Heinrich Loewen, Maxim Janowski, Yadollah Davoudi, Darko Kroepfl

**Affiliations:** ^1^Department of Urology, Pediatric Urology and Urologic Oncology, Kliniken Essen-Mitte, Henricistra*β*e 92, 45136 Essen, Germany; ^2^Institute for Medical Informatics, Biometry and Epidemiology, University of Duisburg-Essen, Hufelandstra*β*e 55, 45122 Essen, Germany; ^3^Die Gesundheitsunion, Urology Health Center, Alter Markt 5-7, 42275 Wuppertal, Germany

## Abstract

We assessed whether changeover from open retropubic [RRP] to robotic-assisted laparoscopic prostatectomy [RALP] means a step forward or backward for the initial RALP patients. Therefore the first 105 RALPs of an experienced open prostatic surgeon and robotic novice—with tutoring in the initial 25 cases—were compared to the most recent 105 RRPs of the same surgeon. The groups were comparable with respect to patient characteristics and postoperative tumor characteristics (all *P* > 0.09). The only disadvantage of RALP was a longer operating time; the advantages were lower estimated blood loss, fewer anastomotic leakages, earlier catheter removal, shorter hospital stay (all *P* < 0.04), and less major complications within 90 days postoperatively (*P* < 0.01). Positive surgical margin rates were comparable both overall and stratified for pT stage in both groups (all *P* < 0.08). In addition, an equivalent number of lymph nodes were removed (*P* > 0.07). Twelve months after surgery, patient reported continence and erectile function were comparably good (all *P* > 0.11). Our study indicates that an experienced open prostatic surgeon and robotic novice who switches to RALP can achieve favorable surgical results despite the initial RALP learning curve. At the same time neither oncological nor functional outcomes are compromised.

## 1. Introduction

While robot-assisted urologic surgery has gained widespread acceptance in the United States with 1,789 installed daVinci surgical systems as of June 30, 2012, it is in its infancy in Europe and in the rest of the world with only 400 and 273 installed units, respectively. Thus, there are still a large number of urologic institutions that are faced with the question if or not a robotic surgical system should be acquired [1]. This question is important since the most frequent robotic procedure, namely, radical prostatectomy, is demanded more and more by the patients, and shows a growing body of literature reporting sound results for the robotic approach both in open-retropubic versus robotic comparative studies [2–20] and in robotic-only studies [21–28]. However, despite the promising results published, implementation of a new surgical technique like robotics in a robotic-naive institution does not automatically guarantee an improved outcome for the patient. Rather, it could even mean an unfavorable outcome in the first patients of the learning curve, if an unfavorable outcome is defined as a worse outcome than that which could have been achieved by the previously established technique [29]. For radical prostatectomy in our and many other institutions the established technique for many years was open radical retropubic prostatectomy (RRP). Thus, when robot-assisted laparoscopic prostatectomy (RALP) was started in our institution in April 2009, we asked ourselves to what extent the initial patients were positively or negatively affected by the new technique. To answer this question we compared the outcomes of RALP and RRP performed by a single surgeon who was a robotic novice but had the greatest experience in open urologic surgery, including prostatectomy, in our institution (KD). To our knowledge, such single surgeon studies focusing on a comparison of the initial RALP cases with a mature series of RRP cases are extremely rare [11, 17]. And so far, only Doumerc et al. have provided a detailed analysis including patient characteristics, surgical data, perioperative course, pathological tumor features, and functional outcome [17]. Yet, it is precisely data of this kind that could help urologists to decide whether to change their prostatectomy policy from an open retropubic approach to a robot-assisted laparoscopic approach. Furthermore, such data can be used in counseling patients who will be part of the initial RALP learning curve.

## 2. Materials and Methods

### 2.1. Patient Selection

Before April 2009, our routine surgical treatment for clinically localized prostate cancer was RRP. Thereafter, following installation of a standard 4-arm daVinci surgical system (Intuitive Surgical Inc., Mountain View, CA) in our institution, all patients with clinically localized prostate cancer were offered RALP as standard surgical treatment. The only reasons for rejection of RALP and recommendation of RRP were contraindications for a steep Trendelenburg position such as previous retinal or cerebral hemorrhage or glaucoma, contraindications for pneumoperitoneum such as severe chronic obstructive lung disease or congestive heart failure, and orthopedic conditions hampering leg spreading. In view of this policy a major selection bias between RALP and RRP patients can be ruled out.

### 2.2. Surgical Intervention

From April 2009 through August 2010, the first 105 consecutive patients underwent RALP performed by KD. During the first 25 operations KD was tutored by an experienced robotic surgeon (YD). The tutoring period of 25 cases was predefined on the basis of previous reports suggesting an initial RALP learning curve of 20–30 cases for experienced open surgeons [30–32]. All procedures were performed using a transperitoneal 6-port approach (4-arm robotic setting) in the technique described by Patel et al. [22] with posterior reconstruction of the rhabdosphincter as described by Rocco et al. [33]. As RRP control we chose the most recent 105 consecutive patients operated on by KD using the technique of Walsh et al. [34] between September 2007 and March 2009. In both groups the decision for or against a nerve sparing technique was made at the discretion of the attending surgeon with due regard to preoperative tumor characteristics and the patient's wishes. Nerve sparing was always done athermally. In the open retropubic series pelvic lymphadenectomy was almost always recommended by the attending surgeon. In the robotic series, however, patient selection for pelvic lymphadenectomy was performed on the basis of the relevant guidelines. A cystographic check of the urethrovesical anastomosis was performed routinely on the 5th postoperative day. If the anastomosis was watertight the catheter was removed the same day, irrespective of the surgical technique.

### 2.3. Data Acquisition

Since March 2004, patient characteristics, clinical and pathological tumor characteristics, surgical data, data on the perioperative course, and postoperative oncological and functional results have been collected prospectively in a Microsoft access database. Therefore followup after discharge from hospital was documented using standardized questionnaires filled in by the patients themselves 3, 6, 12, 18, and 24 months postoperatively. To determine the baseline characteristics the patients were also asked to complete a preoperative questionnaire. Continence status and erectile function were evaluated using the validated ICSmaleSF (short form) questionnaire (questions I1-6 = incontinence symptom domain (ICSmaleIS)) and the validated IIEF-5 questionnaire, respectively [35, 36]. Postoperative complications within 3 months (90 days) after surgery were assessed using the Clavien-Dindo classification [37]. The pathohistological examination of the prostatectomy specimen was performed in a single pathological institute. Positive surgical margins were defined as tumor cells present at the inked surface of the prostate. Lymph nodes inside the lymphadenectomy specimen were identified by digital examination only. Other techniques such as serial sections of the complete specimen or dissolution of the surrounding fatty tissue were not used.

### 2.4. Statistics

In order to validate the comparability of the RALP and RRP groups we first analyzed patient characteristics, clinical and pathological tumor characteristics, and details of surgery. Then both groups were compared with regard to surgical data, data on the perioperative course including relevant complications (Clavien-Dindo grade ≥ IIIa) within 90 days postoperatively, oncological (R-status) and 12-month functional (continence: ICSmaleIS; potency: IIEF-5) results. In this context postoperative erectile function was only analyzed in patients who had a preoperative IIEF-5 score of ≥17, had undergone nerve sparing surgery, and had attempted sexual intercourse postoperatively. Continuous variables are presented as medians with interquartile range (IQR) and categorical variables as frequencies and percentages. We used the two-sided Mann-Whitney *U* test for analysis of continuous variables and the two-sided Fisher's exact test or chi-square test for analysis of categorical variables with two or more characteristics, respectively. The significance level was set at 0.05. Statistical analysis was conducted with the SAS software.

## 3. Results

The RALP and RRP groups were well comparable with respect to patient characteristics (Table 1). With regard to preoperative tumor characteristics we found a slightly but significantly higher prostate-specific antigen (PSA) in patients undergoing RRP. In contrast, there were significantly more palpable tumors in the RALP group (Table 1). No differences were observed between the biopsy Gleason score distributions in the two groups. Likewise, no differences were found with regard to postoperative tumor characteristics (Table 1). Nerve sparing surgery was performed equally often during RRP and RALP. However, pelvic lymphadenectomy was performed significantly less frequently in RALP patients (Table 1).

Perioperative surgical data of both groups are presented in Table 2. The only disadvantage of RALP was a longer operating time. The advantages of RALP were lower estimated blood loss, fewer leakages of the urethrovesical anastomosis on the 5th postoperative day, with resulting earlier catheter removal, and shorter postoperative hospital stay. Furthermore, relevant complications (Clavien-Dindo grade ≥ IIIa) within 90 days postoperatively were significantly less frequent in RALP patients (6.7% versus 20.0%; *P* = 0.0074) (Table 2). This difference was largely driven by a reduction of surgical interventions due to lymphocele formation in the RALP group (Table 2). A detailed list of all Clavien-Dindo grade ≥ IIIa complications is provided in Table 3.

As regards the oncological results we observed R1 rates that were comparable both overall and stratified for pT2, pT3a, and pT3b/4 tumors in both groups (Table 4). In addition, pelvic lymphadenectomy removed an equivalent number of lymph nodes in the context of RALP and RRP (Table 4).

Twelve months after RALP and RRP, patient reported continence and erectile function were comparably good (Table 4).

## 4. Discussion

On the basis of the existing literature there is insufficient evidence to show that changing the prostatectomy policy of an experienced RRP surgeon from RRP to RALP benefits or at least does not harm the initial patients of the RALP learning curve [11, 17]. Moreover, of these two single surgeon comparisons only Doumerc et al. present a detailed analysis including patient characteristics, surgical data, perioperative course, pathological tumor features, and functional outcome [17]. By contrast, White et al. exclusively compare the incidence of positive surgical margins between RALP and RRP performed by a single surgeon, and interpretation of the results is complicated by a surprisingly high rate of R1 findings in pT2 tumors after RRP [11]. The overwhelming majority of publications comparing RALP and RRP outcomes compare either mature RALP series [2, 4, 6, 8, 15, 16, 18, 20] and/or RALP series excluding the first patients of the learning curve [2, 3, 12] and/or series where RALP and RRP were performed by several different surgeons [2, 8–10, 12, 14–16, 18–20]. Although undoubtedly useful in the assessment of RALP and RRP performance in general, the latter studies tell us less about whether changeover of an experienced open prostatic surgeon from RRP to RALP affects the patients of the initial RALP learning curve negatively or positively.

The aim of our case-control study was to examine thoroughly that effect changing the prostatectomy policy this way has on the outcome of patients of the initial robotic learning curve when compared to the most recent patients of a large RRP series from the same surgeon. In other words, we wished to find out to what extent the newly implemented RALP meant an improvement or a step back for the initial patients. The analysis was based on prospectively collected data.

The RALP and the RRP groups were well comparable with respect to the patient characteristics. With respect to preoperative tumor characteristics we found a higher PSA in RRP patients, although a median difference of 1 ng/mL between the two groups is a statistically significant rather than a clinically significant difference. On the other hand, RALP patients were more likely to show palpable tumors, but again the absolute difference was just 4.7%. Analogously, the postoperative tumor characteristics determined from the prostatectomy specimen did not differ between the two groups. Thus, bearing in mind our above mentioned patient selection policy, a major selection bias can be excluded. The largely equal rates of nerve sparing procedures in the two groups (83% versus 86%) were in line with the comparability of the RALP and RRP patients. The lower rate of pelvic lymphadenectomies in RALP patients (65% versus 88%) reflects a change in the decision making process to guideline oriented patient selection when the robotic system was introduced.

Patients in our RALP group benefited from less blood loss and less frequent leakage from the urethrovesical anastomosis on the 5th postoperative day resulting in earlier catheter removal and shorter postoperative hospital stays. The only disadvantage for RALP patients in our analysis was a longer operating time. Although decreased blood loss [2, 3, 5, 7, 9, 12–14, 16, 18, 20] and shorter hospital stays [2, 3, 10, 13, 14, 20] have been reported by several studies, only ours and that of Doumerc et al. already observed these results during the initial robotic learning curve of a single surgeon when compared to his RRP results [17]. This also applies to earlier catheter removal. In addition, only our study and those of Doumerc et al. and Ficarra et al. (analysis of several different surgeons without the first 50 RALP patients) reported this earlier catheter removal in RALP patients despite a standardized cystographic check of the anastomosis on the same postoperative day in the RRP and RALP groups [12, 17]. In further studies which analyzed the duration of catheterization earlier catheter removal was either reported without stating how the decision for catheter removal was made [14], or caused by clearly different approaches in the RRP and RALP groups [2, 19], or statistically not significant [3]. A longer operating time, at least during the initial learning curve, is a well-known disadvantage of the robotic approach in both single-surgeon and multiple-surgeon comparisons [12, 17, 19]. However, a significant reduction in operative time is reported to be achievable by a novice robotic surgeon in the first 41–200 cases already [30, 32, 38]. Indeed, Doumerc et al. demonstrated “3-hour proficiency” after the first 110 cases of a single surgeon [17]. Tewari et al., likewise, observed equivalent operative times of less than three hours in the second 200 RALP patients of a single surgeon when compared to 100 RRP patients of different surgeons from the same institution [2]. 

In our analysis, however, the longer operative time did not translate into a higher rate of postoperative complications. Instead, we already observed a reduction in the rate of relevant complications (Clavien-Dindo IIIa–V) in patients of the initial RALP learning curve. This was primarily due to a significantly lower rate of lymphoceles requiring intervention. This decreased risk of lymphocele formation can be ascribed to the preventive character of the transperitoneal approach [39]. At the same time, however, we retrieved just as many lymph nodes during pelvic lymphadenectomy by the robotic approach as with the open surgical approach. In this context, the relatively high median number of 15 lymph nodes in our RALP patients—retrieved by digital examination alone, without sophisticated techniques such as serial sections of the complete specimen or dissolution of the surrounding fatty tissue—suggests that, contrary to popular opinion, adequate extended pelvic lymphadenectomy is feasible in the robotic learning curve too. This is in line with the results of Di Pierro et al. who reported a median number of 18 lymph nodes in the first 75 RALP patients of a single surgeon series [19].

Furthermore, we demonstrated that despite the initial learning curve the short-term oncologic outcome of RALP patients was not compromised in terms of positive surgical margins. This was also confirmed in subgroup analysis for the different pathological tumor stages pT2, pT3a, and pT3b/4. Indeed, the positive surgical margin rates tended to be lower in our RALP patients in all subgroups, although no statistical significance was seen. It makes sense to choose the positive surgical margin rate as surrogate for the oncologic outcome when comparing different surgical techniques in short-term followup, as this has proved to be the only surgically influenceable independent predictor of progression-free probability [40]. Doumerc and colleagues likewise observed a positive surgical margin rate in the initial RALP learning curve that was comparable to that in RRP patients of the same surgeon. In contrast to our results, however, in their analysis this only held true for pT2 tumors, whereas RALP patients with pT3 tumors showed a significantly higher R1 rate [17]. Nevertheless, to my knowledge, there are at least no comparative studies that report a higher positive surgical margin rate in the subgroup of pT2 tumors alone [3, 8, 11, 12, 14, 17, 19]. Thus, when in doubt, it seems advisable to adopt the policy proposed by Doumerc et al. and, particularly in the first 100 cases of the RALP learning curve, to avoid high-volume tumors which are more likely to be pT3 [17].

Another major point of concern for the patient is the functional outcome. Using the validated ICSmaleSF questionnaire that was filled in by the patients themselves, we found no difference in the ICSmaleIS between RALP and RRP patients 12 months after surgery. Moreover, the median ICSmaleIS of 1.5 (RRP) and 2.0 (RALP) most likely reflects an overall good postoperative continence status of our patients when compared to the median ICSmaleIS of patients with uncomplicated lower urinary tract symptoms associated with benign prostatic enlargement after transurethral prostatic resection (ICSmaleIS 2), noncontact laser therapy (ICSmaleIS 2) or conservative management (ICSmaleIS 3) (data of the CLasP randomized controlled trial) [35]. With regard to the potency outcome, patients with at most mild erectile dysfunction preoperatively who underwent nerve sparing surgery and attempted sexual intercourse postoperatively achieved comparable erectile function 12 months after robotic and open retropubic surgery. Nevertheless, the latter results are limited due to the small case numbers in both groups. Direct comparison of our functional results with those of further comparative studies is not helpful and could even be misleading, since almost all studies apply different methods to evaluate continence and erectile function [2, 3, 10, 12, 14, 17, 19].

One factor, besides the surgeon's extensive experience in pelvic surgery, that was most likely crucial for the favorable results achieved in our robotic cohort despite the initial learning curve was tutoring by a high-volume robotic surgeon during the first 25 cases. The number of 25 cases was predefined on the basis of previous reports suggesting a series of 20–30 cases for experienced open prostatic surgeons to achieve initial proficiency with the robotic system [30–32]. This kind of short mentoring program is also recommended by Kaul et al. from the Vattikuti Urology Institute and by Dev et al. from St John's College of the University of Cambridge in order to optimize the implementation of RALP in a robotically unskilled setting [38, 41].

The limitations of our study are its retrospective nature, the change of procedure for patient selection for pelvic lymphadenectomy in the RALP series, and the short follow up. Its strengths are the prospective data acquisition in an electronic database, the analysis of consecutive patients of a single surgeon, a standardized followup scheme, and the use of validated questionnaires (filled in by the patients themselves) for evaluation of the functional outcome.

Our single-surgeon comparative case-control study indicates that an experienced open prostatic surgeon and robotic novice who switches from RRP to RALP can achieve favorable surgical results, despite the initial RALP learning curve. At the same time oncological and functional outcomes are not compromised. Both the experience of the surgeon and the tutoring during the first 25 RALP cases were most likely crucial for these results. Our data can be especially useful for open prostatic surgeons thinking about switching to the robotic approach as well as for counseling patients who will be part of the initial RALP learning curve of such a surgeon.

## Figures and Tables

**Table 1:** Patient characteristics, preoperative and postoperative tumor characteristics, and details of surgery in the study population—robotic-assisted prostatectomy versus retropubic prostatectomy.

Variable	RRP (*n* = 105)	RALP (*n* = 105)	*P* value
Patient characteristics			

Age (yrs) (median (IQR))	66 (60–70)	67 (61–71)	0.3030
Body mass index (median (IQR))	26.28 (24.42–28.41)	26.37 (24.02–28.41)	0.8551
Prostate weight (g) (median (IQR))	52.4 (42.0–66.0)	55.0 (44.3–67.5)	0.5991
ICSmaleSF incontinence score (median (IQR))	0 (0-1)	1 (0–2)	0.2038

Preoperative tumor characteristics			

PSA (ng/mL) (median (IQR))	7.60 (5.74–11.97)	6.61 (4.90–9.90)	0.0247
Clinical stage (*n* (%))			
T1	70 (66.7)	62 (59.0)	
T2	25 (23.8)	36 (34.3)	
T3	10 (9.5)	2 (1.9)	0.0214
Missing data	0	5 (4.8)	
Biopsy Gleason score (*n* (%))			
5-6	56 (53.3)	61 (58.1)	
7	30 (28.6)	36 (34.3)	
8–10	19 (18.1)	8 (7.6)	0.0728
Missing data	0	0	

Postoperative tumor characteristics			

Pathological stage (*n* (%))			
T2	64 (60.9)	65 (61.9)	
T3a	27 (25.7)	27 (25.7)	
T3b	6 (5.7)	10 (9.5)	0.0923
T4	8 (7.6)	1 (0.9)	
Missing data	0	2 (1.9)	
Prostatectomy Gleason score (*n* (%))			
5-6	39 (37.1)	39 (37.1)	
7a	33 (31.4)	46 (43.8)	
7b	14 (13.3)	9 (8.6)	0.1109
8–10	19 (18.1)	10 (9.5)	
Missing data	0	1 (0.9)	
Lymph node metastasis (*n* (%))			
N0/X	91 (86.7)	96 (91.4)	
N1	13 (12.4)	9 (8.6)	0.3772
Missing data	1 (0.9)	0	
Surgical margin status (*n* (%))			
R0	74 (70.5)	83 (79.0)	
R1	30 (28.6)	21 (20.0)	0.1970
Missing data	1 (0.9)	1 (0.9)	

Details of surgery			

Nerve sparing surgery (*n* (%))			
Yes	89 (84.8)	86 (81.9)	
No	14 (13.3)	18 (17.1)	0.5648
Missing data	2 (1.9)	1 (0.9)	
Pelvic lymphadenectomy (*n* (%))			
Yes	87 (82.8)	68 (64.8)	
No	12 (11.4)	37 (35.2)	<0.001
Missing data	6 (5.7)	0	

IQR: interquartile range; PSA: prostate-specific antigen; RALP: robotic-assisted laparoscopic prostatectomy; RRP: radical retropubic prostatectomy.

**Table 2:** Perioperative surgical data and 90-day postoperative complications—robotic-assisted prostatectomy versus retropubic prostatectomy.

Variable	RRP (*n* = 105)	RALP (*n* = 105)	*P* value
Perioperative surgical data			

Operating time (min) (median (IQR))	185 (172–220)	264 (228–285)	<0.0001
Estimated blood loss (mL) (median (IQR))	600 (400–800)	300 (200–400)	<0.0001
Catheter removal on 5th postoperative day (*n* (%))	65 (61.9)	87 (82.8)	0.0389
Postoperative day of discharge (d) (median (IQR))	8 (7–12)	7 (6–8)	<0.0001

Patients with 90-day postoperative complications (Clavien-Dindo grade ≥ IIIa)			

Clavien-Dindo grade ≥ IIIa (*n* (%))			
Total	21 (20.0)	7 (6.7)	0.0074
Missing data	2 (1.9)	1 (0.9)	
Clavien-Dindo grade ≥ IIIa (*n* (%)) Interventions *due to* lymphoceles (*including only patients with PLA*)	13/87 (14.9)	3/68 (4.4)	0.0359

IQR: interquartile range; PLA: pelvic lymphadenectomy; RALP: robotic-assisted laparoscopic prostatectomy; RRP: radical retropubic prostatectomy.

**Table 3:** Clavien-Dindo grade IIIa–V complications within 90 days postoperatively—robotic-assisted prostatectomy versus retropubic prostatectomy.

Complication	RRP (*n* = 105)*	RALP (*n* = 105)^+^
Clavien-Dindo grade IIIa		

Acute urinary retention with diagnostic cystoscopy	1	0
Suspected melena with gastroscopy	0	1
Symptomatic lymphocele with percutaneous drainage	2	1

Clavien-Dindo grade IIIb		

Symptomatic lymphocele with laparoscopic drainage	7	2
Symptomatic lymphocele with open surgical drainage	4	0
Pelvic hematoma with open surgical drainage	2	1
Superficial wound healing deficit with secondary closure	3	1
Pelvic urinoma with open surgical drainage	1	0
Unilateral hydronephrosis with percutaneous nephrostomy	1	0
Anastomotic stricture with transurethral incision	1	0

Clavien-Dindo grade IVa		

Acute coronary syndrome	1 with cardiac stenting	1 with ICU stay

Clavien-Dindo grade IVb		

Cardiac arrest with successful resuscitation	0	1

Clavien-Dindo grade V		

Death	0	0

Total	23 (in 21 patients)	8 (in 7 patients)

*2 patients presented 2 Clavien-Dindo grade ≥ IIIa complications.

^+^1 patient presented 2 Clavien-Dindo grade ≥ IIIa complications.

ICU: intensive care unit, RALP: robotic-assisted laparoscopic prostatectomy, RRP: radical retropubic prostatectomy.

**Table 4:** 12-month postoperative functional outcome and oncological results stratified for pT stage—robotic-assisted prostatectomy versus retropubic prostatectomy.

Variable	RRP (*n* = 105)	RALP (*n* = 105)	*P* value
12-month functional outcome			

ICSmaleSF incontinence score (median (IQR))	1.5 (0.0–3.0)	2.0 (1.0–5.0)	0.1185
IIEF-5 (median (IQR))	14.5 (7.5–20.5)	17.0 (12.0–23.0)	0.1228

Oncological outcome			

Number of lymph nodes removed (median (IQR))	14 (11–18)	15 (12–20)	0.0739
Surgical margin status, total (*n* (%))			
R0	74 (70.5)	83 (79.0)^#^	
R1	30 (28.6)	21 (20.0)	0.1970
Missing data	1 (0.9)	1 (0.9)	
Surgical margin status, pT2 (*n* (%))			
R0	54 (84.4)	57 (87.7)	
R1	9 (14.1)	8 (12.3)	0.7987
Missing data	1 (1.6)	0	
Surgical margin status, pT3a (*n* (%))			
R0	18 (66.7)	19 (70.4)	
R1	9 (33.3)	8 (29.6)	1.0000
Missing data	0	0	
Surgical margin status, pT3b/4 (*n* (%))			
R0	2 (14.3)	6 (54.5)	
R1	12 (85.7)	5 (45.4)	0.0810
Missing data	0	0	

^#^1 patient with missing pT stage.

RALP: robotic-assisted laparoscopic prostatectomy, RRP: radical retropubic prostatectomy.
